# The Iminosugar UV-4 is a Broad Inhibitor of Influenza A and B Viruses *ex Vivo* and in Mice

**DOI:** 10.3390/v8030071

**Published:** 2016-03-07

**Authors:** Kelly L. Warfield, Dale L. Barnard, Sven G. Enterlein, Donald F. Smee, Mansoora Khaliq, Aruna Sampath, Michael V. Callahan, Urban Ramstedt, Craig W. Day

**Affiliations:** 1 Emergent BioSolutions, Gaithersburg, MD 20879, USA; warfieldk@ebsi.com (K.L.W.); khaliqm@ebsi.com (M.K.); sampatha1@ebsi.com (A.S.); 2 Institute for Antiviral Research, Utah State University, Logan, UT 84322-5600, USA; dale.barnard@usu.edu (D.L.B.); don.smee@usu.edu (D.F.S.); craig.day@usu.edu (C.W.D.); 3 Integrated Biotherapeutics Inc., Gaithersburg, MD 20878, USA; sven@integratedbiotherapeutics.com; 4 Division of Infectious Diseases, Massachusetts General Hospital/Harvard Medical School, Boston, MA 02114, USA; mvcallahan@mgh.harvard.edu; 5 Unither Virology, LLC, Silver Spring, MD 20910, USA; urban_ramstedt@yahoo.com

**Keywords:** Iminosugar, UV-4B, influenza, human bronchial epithelial cells, antiviral, mice

## Abstract

Iminosugars that are competitive inhibitors of endoplasmic reticulum (ER) α-glucosidases have been demonstrated to have antiviral activity against a diverse set of viruses. A novel iminosugar, UV-4B, has recently been shown to provide protection against lethal infections with dengue and influenza A (H1N1) viruses in mice. In the current study, the breadth of activity of UV-4B against influenza was examined *ex vivo* and *in vivo*. Efficacy of UV-4B against influenza A and B viruses was shown in primary human bronchial epithelial cells, a principal target tissue for influenza. Efficacy of UV-4B against influenza A (H1N1 and H3N2 subtypes) and influenza B was demonstrated using multiple lethal mouse models with readouts including mortality and weight loss. Clinical trials are ongoing to demonstrate safety of UV-4B and future studies to evaluate antiviral activity against influenza in humans are planned.

## 1. Introduction

Infection with seasonal, pandemic, and zoonotic influenza viruses can cause significant morbidity and mortality in both healthy and vulnerable populations worldwide. Each year, circulating strains cause three to five million cases of severe influenza, resulting in 250,000 to 500,000 deaths [1]. Influenza remains a major burden despite improved efforts to predict current circulating and antiviral resistant strains, and to develop safe, effective vaccines [2,3]. A number of direct-acting antivirals have been approved for use in the treatment of influenza infections in humans, including the neuraminidase inhibitors peramivir, zanamivir, oseltamivir and the M2 proton channel blockers amantadine and rimantadine. Resistance to these M2-targeted antivirals is widespread and frequently evinced by mutations of one or several bases, or through segmental recombination with resistant strains. Neuraminidase inhibitor-resistant variants have also been shown to quickly arise from within generated virus quasi-species [4,5]. New approaches utilizing host-directed antivirals that do not expose virus to direct selective pressure (as do direct-acting antiviral agents) allow sustained clinically effective therapy against seasonal, pandemic and epizoonotic influenza [4,6,7].

Iminosugars have been described as broad-spectrum antiviral inhibitors [8]. The proposed antiviral mechanism of action for iminosugars is as competitive inhibitors of endoplasmic reticulum (ER) α-glucosidases I and II [9,10]. These are host cell enzymes that remove glucose residues from high-mannose N-linked glycans attached to glycoproteins, allowing for proper protein folding and transport within the cell [11]. Multiple phylogenetically unrelated viruses utilize this cellular pathway to acquire enveloped glycoproteins via ER budding and have been shown to be sensitive to glucosidase inhibition [9]. Inhibition of α-glucosidase I and II is hypothesized to lead to viral glycoprotein misfolding and subsequent transport of the misfolded glycoprotein to the proteosome for degradation and elimination. The iminosugar UV-4, N-(9-methoxynonyl)-1-deoxynojirimycin, is an inhibitor of both glucosidase I and II and has recently been shown to be a potent antiviral drug against dengue (DENV) and influenza viruses *in vivo* [12,13,14]. Recently, we described the efficacy of UV-4B (a hydrochloride salt of UV-4) against lethal infection with a mouse-adapted oseltamivir-sensitive influenza A/Texas/36/91 (H1N1) and an oseltamivir-resistant strain of influenza A/Perth/261/2009 (H1N1) [12]. Additional activity of UV-4B against influenza viruses is described here.

## 2. Materials and Methods

### 2.1. Cytotoxicity Assessment of UV-4B

**Figure 1:**
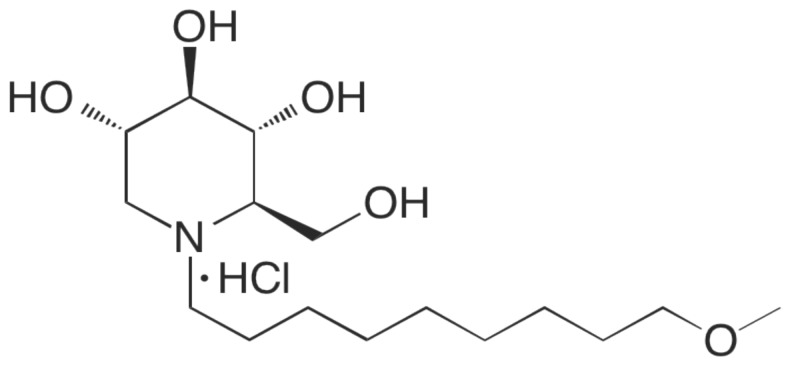
Structure of the iminosugar UV-4B.

The *in vitro* cell cytotoxicity (50% cytotoxic concentration, or CC_50_) of UV-4B, whose structure is shown in Figure 1, was evaluated using EpiAirway™ (Mattek) tissues (primary differentiated normal human bronchial epithelial cells (dNHBE cells)). The tissues were shipped in transwell 96-well plates and used after overnight incubation in the supplied medium. UV-4B was serially diluted 2-fold starting at 500 µM. The transwell insert with the EpiAirway™ tissue and the culture medium were removed from the carrier plate. Fifty (50) µL of the compound dilutions and 200 µL of assay medium were added to the carrier plate. The transwell insert was replaced after rinsing the tissues once with 100 µL of PBS. The tissue was rinsed with 100 µL PBS and the compounds were replaced daily. Cytotoxicity was determined after 72 h using the CellTiter-Glo^®^ kit (Promega). The tissues were rinsed as before and 100 µL of medium were added, followed by 100 µL of the CellTiter-Glo^®^ buffer. Plates were placed on a shaker for two minutes and then incubated for 10 min at ambient temperature. The lysate was transferred to black-walled 96-well plates and luminescence was read in the Tecan reader. Cytotoxicity was determined using untreated tissue as 0% cytotoxic effect and 20% DMSO-treated tissue as 100% cytotoxic effect.

### 2.2. Antiviral Activity in Primary Differentiated Normal Human Bronchial Epithelial (dNHBE) Cells

The virus inoculum was applied to the apical side of each cell insert for 1–2 h, then removed and the apical side washed with 500 uL of HEPES buffer solution. UV-4B, oseltamivir or ribavirin as positive control compounds or media only were applied to both the apical and basal sides of the dNHBE cell system (Mattek (EpiAirway™)) for one hour. The apical medium was removed and the basal side was replaced with fresh compound daily. Virus was harvested from cells after 3 days and samples were cryopreserved. Later, virus yields were determined by endpoint dilution titration in Madin-Darby canine kidney (MDCK) cells. For each concentration, 90% inhibitory concentration (IC_90_) values were determined by regression analysis based on one to three replicate test wells.

### 2.3. Efficacy of UV-4B in Lethal Mouse Models

Groups of 10 BALB/c mice, weighing 17–20 grams at study initiation, were administered doses of 50, 75, 100, or 150 mg/kg by oral gavage TID (eight hour intervals) for 7 to 10 days starting one hour prior to infection. Control groups treated with oseltamivir or vehicle only were included in each study. Under anesthesia, mice were intranasally infected with ~1 LD_90_ of a given virus. Mice were observed for mortality and weight loss for 14 to 21 days. Animals displaying severe illness (>30% weight loss, extreme lethargy, or paralysis) were euthanized. For the *in vivo* studies, concentrations and doses of UV-4B are expressed as the active free base form, UV-4. UV-4B was solubilized in sterile water and administered, 100 µL per dose.

## 3. Results

**Figure 2:**
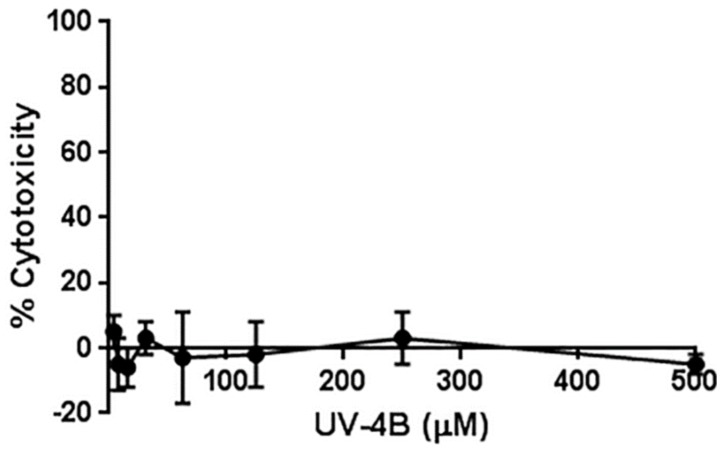
Cytotoxicity of UV-4B in differentiated normal human bronchial epithelial cells (EpiAirway™). Cytotoxic effects of UV-4B were determined in EpiAirway™ tissue on Day 3. An average of four replicates at each concentration are plotted with standard deviation.

The dNHBE cell system has recently gained attention for the ability to recapitulate hallmarks of influenza infections in humans [15,16]. Therefore, dNHBE cells from Mattek (EpiAirway™) were used for cytotoxicity and antiviral evaluations of UV-4B. UV-4B was not cytotoxic at the highest concentrations tested (CC_50_ > 500 μM, Figure 2).

To demonstrate the antiviral effect of UV-4B on a relevant cell type, dNHBE cells infected with influenza A and B viruses were treated with increasing concentrations of UV-4B. Supernatants were collected and virus yield reduction was quantitated by endpoint dilution assays in MDCK cells. The 90% inhibitory concentration (IC_90_) was calculated by the Reed-Muench method. Antiviral activity against influenza A (H3N2) and against some influenza A (H1N1) and influenza B viruses was demonstrated with high concentrations of UV-4B in the primary dNHBE cell culture system with IC_90_ ranging from 82 to >500 µM (Table 1).

**Figure 3:**
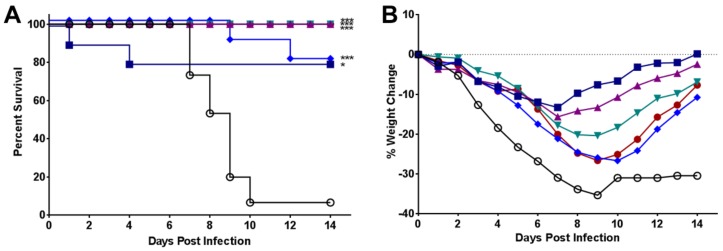

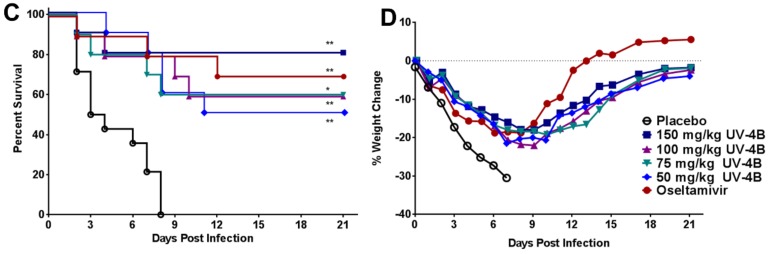
Antiviral efficacy of UV-4B in lethal mouse models of influenza A (H1N1) virus infections. Female BALB/c mice were infected with ~1LD_90_ of influenza virus via intranasal instillation. Mice were administered UV-4B diluted in sterile water. The dose levels are expressed as the active free base form, UV-4. Mice (*n* = 10/group) were orally treated with 50, 75, 100 or 150 mg/kg/dose of UV-4, vehicle or oseltamivir starting one hour before infection. Efficacy data are plotted as percent survival against days post virus exposure. Average weight changes per group are plotted *versus* days post virus exposure. (**A**,**B**) UV-4 efficacy following infection with influenza A/CA/04/09 (H1N1pdm09). In this study, UV-4 was administered for 10 days and oseltamivir was administered at 20 mg/kg/dose BID for 5 days. Mice were monitored for through 14 days post infection. (**C**,**D**) UV-4 efficacy following infection with influenza A/New Caledonia/99 (H1N1). In this study, UV-4 was administered for 7 days and oseltamivir was administered at 10 mg/kg/dose TID for 5 days. Mice were monitored for through 21 days post infection. * *p* < 0.01, ** *p* < 0.001, *** *p* < 0.0001.

Given the broad range of activity of UV-4B against influenza strains, and the fact that we had previously demonstrated that UV-4B protected *in vivo* against lethal infections with the diverse influenza A (H1N1) strains, Texas/36/91 and oseltamivir-resistant Perth/261/2009 (H1N1pdm09); *in vivo* testing against additional influenza viruses was done. Significant improvements in survival following infection with influenza A/California/04/09 (H1N1pdm09) virus [17] were observed in all mice receiving any dose of UV-4B or oseltamivir (*p* < 0.01 to *p* < 0.0001, Figure 3A). Mice in the 50 mg/kg/dose group died significantly later in the infection, at an average of 10.5 days, as compared to the placebo group (~8 days, *p* < 0.01, Figure 3A). The minimum effective dose (MED) of UV-4B that led to 100% survival against influenza A/California/04/2009 (pdm H1N1) was 75 mg/kg and the 50% MED was less than the lowest dose tested (50 mg/kg, 80% survival). All mice infected with influenza A/California/04/09 (H1N1 pdm09) virus lost weight (placebo, UV-4B or oseltamivir, Figure 3B). All doses of UV-4B significantly protected against weight loss from Days 4–7 compared to placebo-treated mice (*p* < 0.05 to *p* < 0.0001). Amelioration of weight loss by UV-4B was comparable to oseltamivir treatment (*p* < 0.01 to *p* < 0.0001). For mice treated with UV-4B at 75, 100, or 150 mg/kg/dose, significant weight loss protection extended to Day 14 post-virus exposure (*p* < 0.05 to *p* < 0.0001). This study showed that all doses of UV-4B were effective in significantly ameliorating weight loss for the entire experiment, in prolonging mean time to death, and in promoting survival. BALB/c mice infected with influenza A/New Caledonia/20/99 [18] showed 80% survival when treated with 150 mg/kg TID of UV-4B, while 0% survived in placebo (*p* < 0.001, Figure 3C). The survival curve (Figure 3C) showed a significant (*p* < 0.01) dose-related response with UV-4B. Weight loss was observed in all infected test groups, but was significantly ameliorated in mice treated with UV-4B or oseltamivir compared with placebo (Figure 3D). The weight loss in animals treated with all doses of UV-4B was similar to that of oseltamivir-treated groups up to Day 8 or 9 post-virus exposure, with recovery faster in the oseltamivir-treated group (Figure 3D). Complete (100%) protection from lethal influenza A/New Caledonia/20/99 (H1N1) virus disease was not observed in this study. The highest dose of 150 mg/kg provided 80% survival and the 50% MED for lethality was 50 mg/kg (Figure 3C).

**Figure 4:**
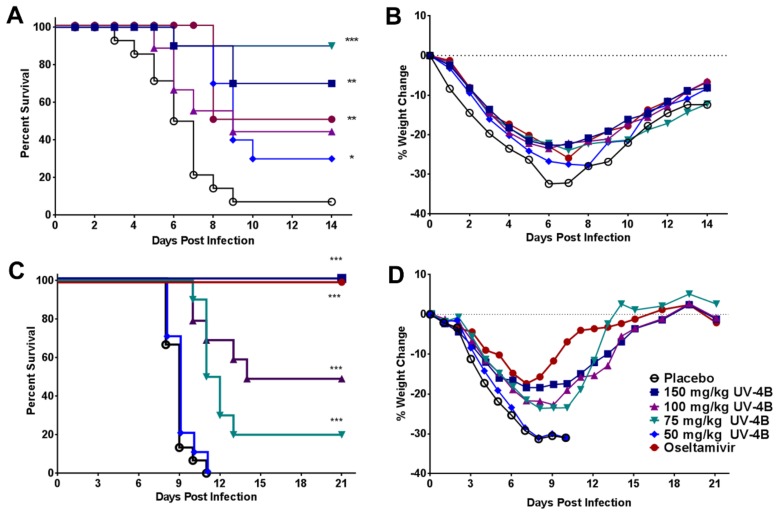
Antiviral efficacy of UV-4B in lethal mouse models of influenza A (H3N2) and B virus infections. Female BALB/c mice (*n* = 10/group) were infected with ~1LD_90_ of influenza virus via intranasal instillation. Mice were administered UV-4B diluted in sterile water. The dose levels are expressed as the active free base form, UV-4. Mice were orally treated with 50, 75, 100 or 150 mg/kg/dose of UV-4, vehicle or oseltamivir starting one hour before infection. Efficacy data are plotted as percent survival against days post virus exposure. Average weight changes/group are plotted *versus* days post virus exposure. (**A**,**B**) UV-4 efficacy following infection with influenza A/PA/10/2010 (H3N2) swine virus. In this study, UV-4 was administered for 7 days and oseltamivir was administered at 20 mg/kg/dose BID for 5 days. Mice were monitored for through 14 days post infection. (**C**,**D**) UV-4 efficacy following infection with influenza B/Sichuan/279/99. In this study, UV-4 was administered for 10 days and oseltamivir was administered at 10 mg/kg/dose TID for 5 days. Mice were monitored for through 21 days post infection. * *p* < 0.01, ** *p* < 0.001, *** *p* < 0.0001

The efficacy of UV-4B was also tested in a lethal mouse model of influenza A/Pennsylvania/10/2010 (H3N2) swine variant strain virus [19]. The most effective dose of UV-4B in promoting survival of infected mice was 75 mg/kg (90% survival, Figure 4A). The 50, 100, and 150 mg/kg doses of UV-4B were not as effective as the 75 mg/kg dose; survival levels in these groups were 30%, 50% and 70%, respectively (Figure 4A). Significant differences in survival were noted for mice treated with UV-4B at dose levels of 150 (*p* = 0.0003), 75 (*p* < 0.0001) and 50 (*p* = 0.0053) mg/kg but not at the 100 mg/kg dose level (*p* = 0.0844). Fifty percent (50%) of mice survived when treated with oseltamivir (*p* = 0.0003); deaths in this group occurred only on Day 8 post-virus exposure; mice administered UV-4B at dose levels ≥75 mg/kg had similar levels of protection (≥50%) to oseltamivir-treated mice (Figure 4A). Untreated, infected mice experienced far greater weight loss and demonstrated more morbidity than did mice receiving UV-4B at all doses or oseltamivir until after Day 9 post-virus exposure (Figure 4A,B). The amelioration of weight loss in treated groups was significant (*p* < 0.05 to *p* < 0.01) for all treated mice at Days 1 and 2 post-infection (Figure 4B). UV-4B treatment at 50 and 150 mg/kg/dose and treatment with oseltamivir, consistently prevented the significant weight loss observed in untreated mice through Day 6 (*p* < 0.05 to *p* < 0.0001). Between Days 7 and 8, mice in all groups began recovery from weight loss, although no group returned to their starting weight by the end of the experiment, Day 14 (Figure 4B).

BALB/c mice infected with influenza B/Sichuan/379/99, Yamagata lineage [18] were fully protected from death when treated with UV-4B at 150 mg/kg (Figure 4C). Mice treated with 100 mg/kg survived significantly better (50% survival) than did the placebo-treated mice (0% survival) (Figure 4C). A significant survival benefit was also observed in the group of mice treated with 75 mg/kg of UV-4B, while survival results in mice treated with 50 mg/kg were nearly identical with the placebo control (Figure 4C). Weight loss was observed in all infected test groups, but was significantly less in mice treated with ≥75 mg/kg of UV-4B or oseltamivir (Figure 4D). A clear dose-related response with increased survival benefit was observed. The 100% MED for UV-4B against the influenza B/Sichuan/379/99 strain was 150 mg/kg of UV-4B and the 50% MED was 100 mg/kg of UV-4B (Figure 4C).

## 4. Discussion

In the current studies, antiviral activity of UV-4B against influenza A and B viruses was shown in primary dNHBE cells, the principal target tissue for influenza. In this *ex vivo* cell culture system, inhibition of replication of influenza A (H1N1, H3N2) and influenza B strains was demonstrated at high concentrations (82 to > 500 μM). Our findings agree with previous glucosidase inhibitor studies with castanospermine, bromoconduritol, *N-*butyl-deoxynojirimycin and *N-*nonyl-deoxynojirimycin where viral replication inhibition was variable between virus subtypes and strains. In these previous studies, strain- and cell type-dependent inhibition occurred despite clear effects on glycosylation of influenza virus structural proteins [20,21,22,23,24,25]. Using single-dose pharmacokinetics such as reported in [13], modeling of the pharmacokinetics expected on a three-times-daily dosing schedule at the efficacious doses reported here predicts *in vivo* trough plasma levels in the low micromolar range. This is 25- to 150-fold lower than the *in vitro* IC_90_ values, indicating that for UV-4B the *in vitro* IC_90_ values are not a reliable predictor of pharmacodynamic activity.

In addition to broad *ex vivo* antiviral activity using dNHBE cells, the efficacy of UV-4B against influenza A (H1N1 and H3N2 subtypes) and influenza B has now been demonstrated using multiple lethal mouse models. The efficacy of UV-4B was evaluated against lethal infections with mouse-adapted H1N1 subtypes of influenza including A/Texas/36/91 [12], A/California/04/2009 (Figure 3A,B), and A/New Caledonia/20/99 (Figure 3C,D) as well as an oseltamivir-resistant strain of influenza A/Perth/261/2009 [12]. The frequency, MED, and therapeutic window were determined based on survival analysis in the mouse-adapted influenza A/Texas/36/91 (H1N1) model [12]. UV-4B treated mice were also tested for virus titers in serum and lung tissues and cytokine responses in the serum of infected mice [12]. The 100% MED for UV-4B in lethal mouse models of influenza A (H1N1) appears to be 75–150 mg/kg when given TID orally. Treatment can be initiated as late as 72 h after infection to achieve a statistically significant survival benefit with UV-4B treatment [12]. Additional studies were performed to demonstrate antiviral activity against influenza A (H3N2) and B viruses in lethal mouse models.

Previous work has demonstrated efficacy against a broad range of dengue viruses *in vitro* and *in vivo* [13,14]. The *in vitro* concentrations and *in vivo* dosing regimens required for antiviral activity against dengue virus appear to be lower than those required for efficacy against influenza. In a dengue mouse model of antiviral efficacy for UV-4, lower doses are required for complete protection from lethal disease (10–20 mg/kg) [13,14]. The 50% inhibitory concentration (IC_50_) values for UV-4B were recently shown to range from 2.10 µM (DENV1 SH29177) to 86.49 µM (DENV3 H87) amongst isolates from the four serotypes that were tested. Taken together, our data establish that UV-4B is a broad inhibitor of both dengue and influenza viruses. UV-4B is currently in Phase 1 clinical trials [26] where it has been found to be well-tolerated as single oral doses in healthy volunteers. Future safety and efficacy studies of UV-4B against dengue and influenza in humans are planned.

## Figures and Tables

**Table 1:** Summary of UV-4B activity against multiple influenza strains in Differentiated Normal Human Bronchial Epithelial (dNHBE) cells (EpiAirway™).

Virus Strain	MOI	UV-4B IC_90_	Control	IC_90_
**A/California/07/09 (H1N1)**	10^−2^ CCID_50_/cell	>320 µM ^a,e^	Oseltamivir	0.36 µM
**A/California/12/2012 (H1N1)**	10^−4^ CCID_50_/cell	320 µM ^c^	Ribavirin	<13 µM ^b^
	10^−3.5^ CCID_50_/cell	219 µM ^e^		4.7 µM
**A/Victoria/3/75 (H3N2)**	10^−4^ CCID_50_/cell	440 µM ^c^	Ribavirin	<13 µM ^b^
	10^−3.5^ CCID_50_/cell	483 µM ^e^		5.4 µM
**A/Texas/50/2012 (H3N2)**	10^−5^ CCID_50_/cell	82 µM ^d^	Ribavirin	<13 µM ^b^
**B/Brisbane/60/2008**	10^−2^ CCID_50_/cell	200 µM ^e^	Oseltamivir	3.0 µM
**B/Florida/4/2006**	10^−2^ CCID_50_/cell	150 µM ^e^	Oseltamivir	3.4 µM
**B/Massachusetts/2/2012**	10^−4^ CCID_50_/cell	209µM ^c^	Ribavirin	<13 µM^b^
	10^−3.5^ CCID_50_/cell	245 µM ^c^		4.2 µM
**B/Malaysia/2506/2004**	10^−4^ CCID_50_/cell	>500 µM ^a,^^c^	Ribavirin	<13 µM^b^
	10^−3.5^ CCID_50_/cell	>500 µM ^a,e^		4.8 µM

^a^ The IC_90_ was greater than the highest concentration tested; ^b^ The IC_90_ was less than the lowest concentration tested (13 µM); ^c^ Single replicate well per test concentration; ^d^ Duplicate test wells in a single assay; ^e^ Triplicate test wells in a single assay.

## References

[1] Influenza (seasonal), Fact Sheet no211, March 2014. http://www.who.int/mediacentre/factsheets/fs211/en/.

[2] Watanabe T., Watanabe S., Maher E.A., Neumann G., Kawaoka Y. (2014). Pandemic potential of avian influenza a (h7n9) viruses. Trends Microbiol..

[3] Pascua P.N., Choi Y.K. (2014). Zoonotic infections with avian influenza a viruses and vaccine preparedness: A game of “mix and match”. Clin. Exp. Vaccine Res..

[4] Loregian A., Mercorelli B., Nannetti G., Compagnin C., Palu G. (2014). Antiviral strategies against influenza virus: Towards new therapeutic approaches. Cell. Mol. Life Sci..

[5] Hurt A.C., Hardie K., Wilson N.J., Deng Y.M., Osbourn M., Leang S.K., Lee R.T., Iannello P., Gehrig N., Shaw R. (2012). Characteristics of a widespread community cluster of h275y oseltamivir-resistant a(h1n1)pdm09 influenza in Australia. J. Infect. Dis..

[6] Zumla A., Memish Z.A., Maeurer M., Bates M., Mwaba P., Al-Tawfiq J.A., Denning D.W., Hayden F.G., Hui D.S. (2014). Emerging novel and antimicrobial-resistant respiratory tract infections: New drug development and therapeutic options. Lancet Infect. Dis..

[7] Lee S.M., Yen H.L. (2012). Targeting the host or the virus: Current and novel concepts for antiviral approaches against influenza virus infection. Antivir. Res..

[8] Zitzmann N., Block T., Methta A., Rudd P., Burton D., Wilson I., Platt F., Butters T., Dwek R.A. (2005). Glycosylation: Disease targets and therapy. Adv. Exp. Med. Biol..

[9] Chang J., Block T.M., Guo J.T. (2013). Antiviral therapies targeting host er alpha-glucosidases: Current status and future directions. Antivir. Res..

[10] Dalziel M., Crispin M., Scanlan C.N., Zitzmann N., Dwek R.A. (2014). Emerging principles for the therapeutic exploitation of glycosylation. Science.

[11] Helenius A., Aebi M. (2004). Roles of n-linked glycans in the endoplasmic reticulum. Annu. Rev. Biochem..

[12] Stavale E.J., Vu H., Sampath A., Ramstedt U., Warfield K.L. (2015). *In vivo* therapeutic protection against influenza a (h1n1) oseltamivir-sensitive and resistant viruses by the iminosugar uv-4. PLoS ONE.

[13] Perry S.T., Buck M.D., Plummer E.M., Penmasta R.A., Batra H., Stavale E.J., Warfield K.L., Dwek R.A., Butters T.D., Alonzi D.S. (2013). An iminosugar with potent inhibition of dengue virus infection *in vivo*. Antivir. Res.

[14] Warfield K.L., Plummer E., Sayce A.C., Alonzi D., Tang W., Tyrrell B.E., Hill M.L., Caputo A.T., Killingbeck S.S., Beatty P.R. (2016). Inhibition of endoplasmic reticulum glucosidases is required for *in vitro* and *in vivo* dengue antiviral activity by the iminosugar uv-4. Antivir. Res..

[15] Triana-Baltzer G.B., Babizki M., Chan M.C., Wong A.C., Aschenbrenner L.M., Campbell E.R., Li Q.X., Chan R.W., Peiris J.S., Nicholls J.M. (2010). Das181, a sialidase fusion protein, protects human airway epithelium against influenza virus infection: An *in vitro* pharmacodynamic analysis. J. Antimicrob. Chemother..

[16] Davis A.S., Chertow D.S., Moyer J.E., Suzich J., Sandouk A., Dorward D.W., Logun C., Shelhamer J.H., Taubenberger J.K. (2015). Validation of normal human bronchial epithelial cells as a model for influenza a infections in human distal trachea. J. Histochem. Cytochem..

[17] Smee D.F., Barnard D.L. (2013). Methods for evaluation of antiviral efficacy against influenza virus infections in animal models. Methods Mol. Biol..

[18] Smee D.F., Wong M.H., Bailey K.W., Sidwell R.W. (2006). Activities of oseltamivir and ribavirin used alone and in combination against infections in mice with recent isolates of influenza a (h1n1) and b viruses. Antivir. Chem. Chemother..

[19] Julander J.G., Kesler K., Van Wettere A.J., Morrey J.D., Smee D.F. (2014). The use of plethysmography in determining the severity of lung pathology in a mouse model of minimally lethal influenza virus infection. Antivir. Res..

[20] Saito T., Yamaguchi I. (2000). Effect of glycosylation and glucose trimming inhibitors on the influenza a virus glycoproteins. J. Vet. Med. Sci..

[21] Pan Y.T., Hori H., Saul R., Sanford B.A., Molyneux R.J., Elbein A.D. (1983). Castanospermine inhibits the processing of the oligosaccharide portion of the influenza viral hemagglutinin. Biochemistry.

[22] Karaivanova V.K., Luan P., Spiro R.G. (1998). Processing of viral envelope glycoprotein by the endomannosidase pathway: Evaluation of host cell specificity. Glycobiology.

[23] Datema R., Romero P.A., Rott R., Schwarz R.T. (1984). On the role of oligosaccharide trimming in the maturation of sindbis and influenza virus. Arch. Virol..

[24] Romero P.A., Datema R., Schwarz R.T. (1983). N-methyl-1-deoxynojirimycin, a novel inhibitor of glycoprotein processing, and its effect on fowl plague virus maturation. Virology.

[25] Hussain S., Miller J.L., Harvey D.J., Gu Y., Rosenthal P.B., Zitzmann N., McCauley J.W. (2015). Strain-specific antiviral activity of iminosugars against human influenza a viruses. J. Antimicrob. Chemother..

[26] Nct02061358: Randomized, Double-Blind, Placebo-Controlled, Parallel Group, Single-Ascending Dose Study to Determine the Safety, Tolerability and Pharmacokinetics of uv-4b Solution Administered Orally in Healthy Subjects. https://clinicaltrials.gov/ct2/show/NCT02061358.

